# Outbreaks of thrush in pigeons in Punjab State of India

**DOI:** 10.1007/s00580-014-1958-y

**Published:** 2014-07-08

**Authors:** Madhav Mugale, Abid Ali Bhat, D. S. Gavhane, Sartaj Ahmad Bhat

**Affiliations:** Teaching Veterinary Clinical Complex, Khalsa College of Veterinary and Animal Sciences, Amritsar, Punjab India

**Keywords:** Candidiasis, Cytology, Histopathology, Pigeons, Thrush

## Abstract

Due to high monetary turnover in business, white pigeon keeping for game purposes is gaining more popularity in Punjab. Overcrowding and poor management by undertrained naive farmers make these birds more susceptible to diseases not known so far in this region. A farmer reported that about a hundred pigeons were unable to feed properly and regurgitate feed. Birds lost body condition gradually, and three among these died. Both alive and dead pigeons were presented to the Veterinary Clinical Complex (VCC) for detailed examination. All these pigeons were found to be cachectic with wasting of breast muscles. On necropsy, no significant gross lesions were recorded in most of the visceral organs, except mottling of the liver. However, in the oral cavity, gray Turkish towel-like lesions were seen at the opening of the pharynx which continued into the larynx and proximal esophagus. Microscopic examination of material scrapped from lesions revealed a large number of budding yeast-like organisms and pseudohyphae, suggestive of *Candida* spp. Histologically, marked necrosis and sloughing of oral and esophageal mucosal epithelium with the presence of pyogranulomatous inflammation containing a large number of *Candida* organism were observed. To the authors’ knowledge, there seems to be no outbreak of thrush in pigeons in Punjab previously.

## Introduction

Candidiasis is an occasional opportunistic fungal disease of importance in poultry. It has also been reported to be a disease or an intestinal infection in numerous species of wild birds that are being raised in captivity (Milton 1999). The disease is sporadic in nature, and outbreaks occur where proper management protocols are not followed. *Candida albicans* (*C. albicans*), a yeast-like fungus, is the primary cause of candidiasis. *C. albicans* is a common environmental organism and an opportunistic pathogen having normal inhabitant of the avian crop. Candidiasis has been observed in chickens, pigeons, turkeys, geese, guinea fowl, pheasants, quail, parrots, and other birds (Moretti et al. 2000). Ingestion of contaminated food or drinking water is the usual means for its transmission. Contaminated environments, such as litter from poultry and game bird rearing facilities, refuse disposal areas, discharge sites for poultry operations, and areas contaminated with human waste are suggested as sources for *Candida* exposure for birds. Impacted food, beak abnormalities, and tongue injuries predispose the bird to oral candidiasis. Young birds with crop stasis are especially more susceptible to this disease (Bauck 1994; Kunkle 2003).

The natural mode of feeding for nestling pigeons is by thrusting their soft, pliable beak into the parent’s gullet to drink crop milk secreted from the lining epithelium of the crop (Tottenham 1982); therefore, candida organisms can be transmitted from infected parents to their nestlings through crop milk. Candidiasis is almost always secondary to other diseases or due to the immunosuppression or after prolonged antibiotic therapy. In birds, particularly, the stress of heavy flight or force racing or force feeding (Tsat et al. 1994) may cause oral or gastrointestinal candidiasis. If not treated on proper time, this may lead to mortality. This disease has tremendous zoonotic importance for humans. The present manuscript describes the outbreaks of oral candidiasis, its diagnosis, pathological features, and therapeutic management.

## Case description

Two outbreaks were reported near Ludhiana District, Punjab, of about 120 and 250 white game pigeons each. The affected pigeons suffered from inappetence, anorexia, and regurgitating feed. Bird’s feather quality became poor and unkempt. Among the affected birds, a few were reported of having diarrhea. The affected birds lost their body condition gradually and became cachectic with wasting of breast muscles. In the first and second outbreaks, three and two pigeons died, respectively. The representative live and dead pigeons were presented to investigate the cause. History taken from owner was noted. The dead birds were subjected to necropsy.

**Fig. 1:**
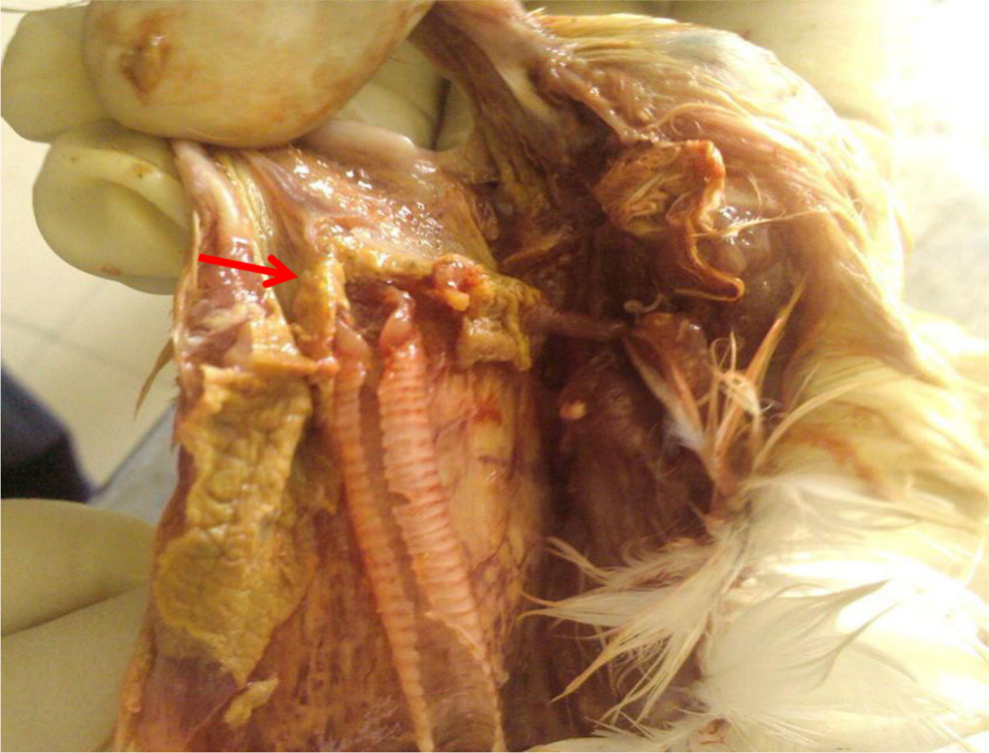
Pseudomembrane on esophagus, pharynx, and larynx (*red arrow*)

At necropsy, no significant gross lesions were recorded in most visceral organs, except the liver. The liver showed mottling. Thickening of the crop mucosae were noted. However, on opening the oral cavity, gray towel-like lesions at the opening of the pharynx and larynx were seen, and this yellowish white pseudomembrane on the mucosa continued into the proximal esophagus (Fig. 1). Impression smears were made from lesions of oral and esophageal mucosa. Suitable pieces of tissue were collected in 10 % neutral buffered formalin and subjected to routine histopathological examination.

**Fig. 2:**
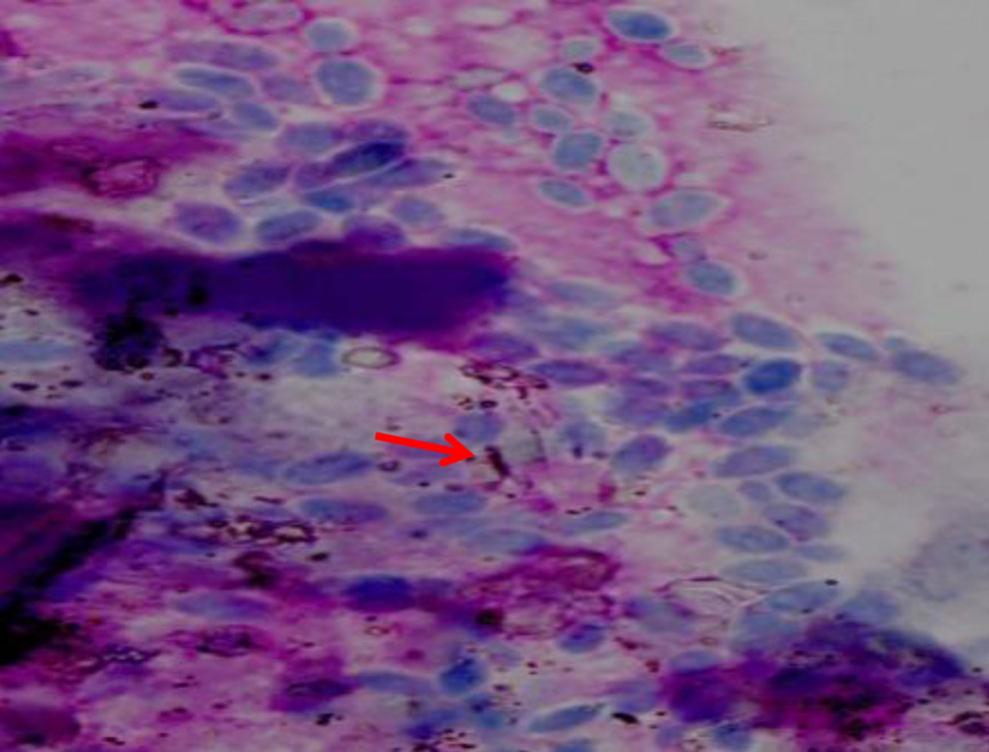
Large number of yeast-like organisms and early pseudohyphae (*red arrow*)

Cytological examination impression smears stained by Leishman’s stain revealed a large number of budding yeast-like organisms and early pseudohyphae (Fig. 2), suggesting *Candida* as an etiological agent and, hence, the disease thrush.

**Fig. 3:**
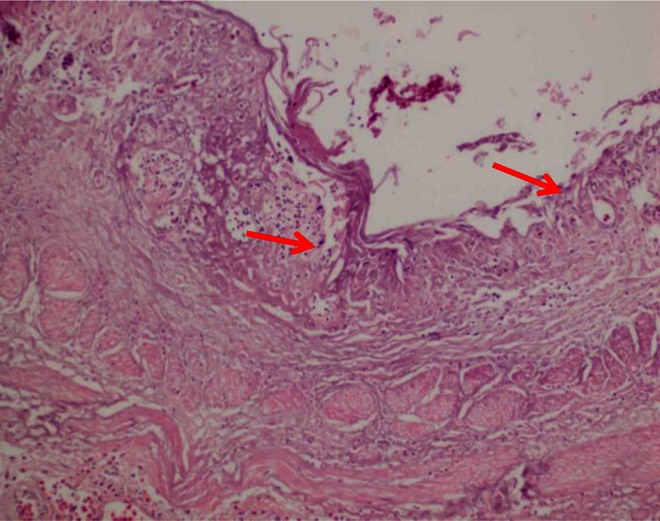
Section of esophagus showing marked sloughing of epithelium and inflammation (*red arrow*)

**Fig. 4:**
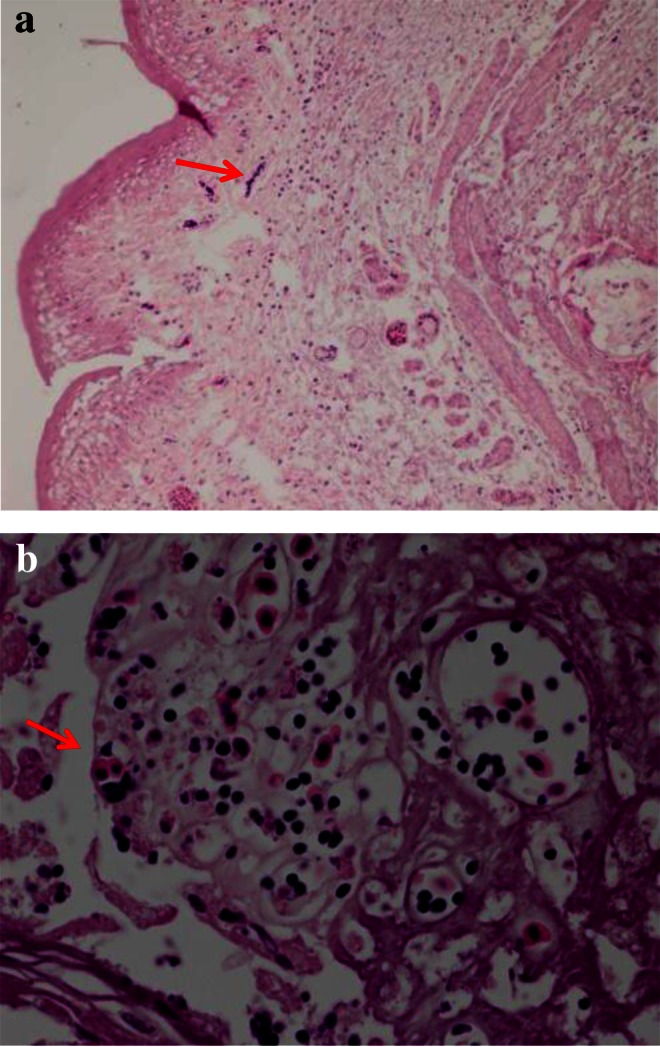
**a** Section of esophagus showing hyperkeratosis and candida organism (*red arrow*, ×10). **b** Higher magnification of **a** showing division of and candida organism (*red arrow*, H & E, ×40)

Histopathologically, marked necrosis and hyperkeratosis of oral and esophageal mucosa were observed. Erosion and ulcerations along with the presence of mild pyogranulomatous inflammation containing a large number of *Candida* organisms were observed in the oral and esophageal mucosa (Figs. 3 and 4a, b).

On the basis of gross and histopathological features, the disease was diagnosed as oral *candidiasis*, i.e., thrush.

All the affected birds from the farm were treated with copper sulfate at 2 g/l of water for 15 days. Farmers were suggested oral application of candid lotion to the affected birds, which showed dramatic recovery from the infection.

## Discussion

*C. albicans* is a saprophytic fungus and commensal of the upper avian digestive tract (Bauck 1994). It can be readily isolated from the intestine and mucocutaneous surfaces of birds, animals, and humans. Pathology due to infection is an aberration brought on by a lapse of immunologic homeostasis or shifts in the ecology of microflora colonizing the host (Kunkle 2003). Increased virulence of the fungus plays a vital role in establishing the disease (Chute 2001). Litter from poultry houses and game bird areas, waste, and disposal areas contaminated with human waste are suggested as potential sources for exposure to *Candida* introduction (Bauck 1994; Oglesbee 1997). The risk factors, which predispose to candidiasis and aggravate disease, include malnutrition, vitamin D deficiency, poor hygiene, prolonged use of antibiotic causing suppression of normal bacterial flora, and stress leading to immunosuppressive diseases (Velasko 2000). In the present case, gross examination revealed gray towel-like lesions at the opening of the pharynx and larynx, which has also been reported by many workers (Schmidt et al. 2003; Velasko 2000; Bauck 1994). On histopathological examination, extensive tissue damage of the upper digestive tract was observed. This damage might be due to various recognized virulence factors of *C. albicans* such as adhesins having affinity for fibronectin on the cell surfaces and phospholipase present on hyphal tips enhancing invasiveness and yeast forms causing tissue damage (Macdonald 1984; Ruchel 1984). *C. albicans* can produce proteinases by which keratin and collagenase are lysed (Hattori et al. 1984; Negi et al. 1984). The presence of hyperkeratosis in the birds might partially be due to mechanical irritation by the impacted food. Endotoxin produced by *C. albicans* might have played a role in the development of hyperkeratotic lesions (Tsai et al. 1992; Ruchel 1984). Classically, this disease is associated with either vitamin A deficiency or prolonged antibiotic administration. The source of *candida* infection in the present outbreaks could not be ascertained. However, the crop milk supplied by the parents might be one of the several reasons. The lack of antibody such as IgA and a high content of carbohydrates in the crop might have favored the establishment of *candidial* infection. In Punjab, there is a trend among farmers, i.e., self-treatment in animals as well as in avian species, particularly with antibiotics which are easily available; hence, their prolonged use might have favored the establishment of candida infection in these pigeons. Lack of knowledge, overcrowding, and poor management by undertrained naive farmers are other major precipitating factors.

## Conclusion

The source of infection in the present outbreak could not be ascertained. However, there are many factors which play a role in the development of candidiasis. All these factors deserve further study in relation to this fungal infection in young pigeons.
